# Construction and characterization of a new TRAIL soluble form, active at picomolar concentrations

**DOI:** 10.18632/oncotarget.25519

**Published:** 2018-06-05

**Authors:** Matias Eliseo Melendez, Renato José Silva-Oliveira, Anna Luiza Silva Almeida Vicente, Lidia Maria Rebolho Batista Arantes, Ana Carolina de Carvalho, Alberto Luis Epstein, Rui Manuel Reis, André Lopes Carvalho

**Affiliations:** ^1^ Molecular Oncology Research Center, Barretos Cancer Hospital, Barretos, São Paulo, Brazil; ^2^ UMR1179, INSERM-UVSQ, Handicap Neuromusculaire, Biotherapie et Pharmacologie Appliquées, Université de Versailles-Saint Quentin en Yvelines, Versailles, France; ^3^ Life and Health Sciences Research Institute (ICVS), Health Sciences School, University of Minho, Braga, Portugal; ^4^ ICVS/3B’s-PT Government Associate Laboratory, Braga/Guimarães, Portugal

**Keywords:** TRAIL, apoptosis, cancer treatment, amplicon vectors

## Abstract

Apoptosis induction has emerged as a treatment option for anticancer therapy. Tumor necrosis factor-related apoptosis-inducing ligand (TRAIL), a type II transmembrane protein, is a potent and specific pro-apoptotic protein ligand, which activates the extrinsic apoptosis pathway of the cell death receptors. Here we describe the construction and characterization of a new soluble TRAIL, sfTRAIL, stabilized with the trimerization Foldon domain from the Fibritin protein of the bacteriophage T4. Supernatants of 0.22 μM-filtered supernatants were produced in Vero-transduced cells with HSV1-derived viral amplicon vectors. Experiments were undertaken in two known TRAIL-sensitive (U373 and MDA.MB.231) and two TRAIL-resistant (MCF7 and A549) cell lines, to determine (i) whether the sfTRAIL protein is synthetized and, (ii) whether sfTRAIL could induce receptor-mediated apoptosis. Our results showed that sfTRAIL was able to induce apoptosis at concentrations as low as 1899.29 pg/mL (27.71 pM), independently of caspase-9 activation, and reduction in cell viability at 998.73 fM.

## INTRODUCTION

Apoptosis induction has emerged as a treatment option in anticancer therapy. Different from non-apoptotic forms of cell death, apoptosis does not result in the release of cellular pro-inflammatory molecules into the tumor microenvironment, which may lead to further tissue damage [1]. Apoptosis is initiated by two different mechanisms: the death receptor-mediated (extrinsic pathway) and the mitochondria-triggered one (intrinsic pathway). Over the last decade, activation of extrinsic pathway of apoptosis has been considered an attractive therapeutic strategy to promote apoptosis of tumor cells [2–4]. Among the death receptors explored for cancer treatment, receptors belonging to the pro-apoptotic pathway of the TNF-related apoptosis-inducing ligand (TRAIL) has gained interest and even entered in clinical trials in combination with cytotoxic chemotherapy.

Tumor necrosis factor-related apoptosis-inducing ligand (TRAIL), a type II transmembrane protein is a potent and specific pro-apoptotic protein ligand, able to bind to five different receptors. Upon trimerization, TRAIL promotes apoptosis by binding to their effector death receptors, TRAIL-R1 and TRAIL-R2, triggering the recruitment of the adaptor molecule Fas-associated death domain and procaspase 8 to the cytoplasmic death domain of the receptor [5, 6]. Activated caspase-8 then cleaves caspase-3, which in turn promotes cleavage of critical downstream cellular proteins, finally leading to DNA fragmentation and cell death. Decoy receptor 1 (TRAIL-R3), decoy receptor 2 (TRAIL-R4) and the soluble receptor osteoprotegerin lack active cytoplasmic death domains, blocking the apoptotic machinery [7].

TRAIL-induced apoptosis leaves non-tumoral cells unharmed, underlying its potential as a candidate therapeutic protein in the carcionogenesis cascade [2, 8]. While this oncotargeted potential is poorly understood, it has made TRAIL pathway an attractive target for cancer treatment, leading to the development of clinical trials of recombinant human TRAIL proteins. Among the tumor types already tested in clinical trials, Non-Small Cell Lung Cancer (Phase II), Colorectal Cancer (Phase I) and Non-Hodgkin’s Lymphomas (Phase I). Although, despite the therapeutic potential of cell death receptor agonists already tested in preclinical models, the translation of these effects into the clinic remains disappointing, mainly due to protein instability and high cost of production. Here, we describe the construction and characterization of a novel recombinant soluble TRAIL protein, named sfTRAIL stabilized with the trimerization Foldon domain, showing its capabilities of inducing robust apoptosis, even at picomolar concentrations.

## RESULTS

### Expression of TRAIL receptors

We further determined the expression of TRAIL receptors, TRAIL-R1 and TRAIL-R2, in A549, MCF7, U373 and MDA.MB.231 cell lines by flow cytometry (Supplementary Figure 1). Resistance/sensitivity to TRAIL-mediated apoptosis was previously described for these cell lines [9–12]. Both sensitive cell lines (U373 and MDA.MB.231) showed higher expression of TRAIL-R1 and TRAIL-R2 than the MCF7 cell line. Interestingly, the A549 cell line showed high expression of both TRAIL receptors.

### Sensitivity to rhTRAIL induced apoptosis

In order to analyze the sensitivity and resistance of cell lines to TRAIL induced apoptosis, commercially available rhTRAIL was used (Supplementary Figure 2). Both TRAIL-sensible cell lines, U373 and MDA.MB.231 showed an increased apoptosis, when compared to control groups. Interestingly, MCF7 cell line also showed an increase in apoptosis. Only A549 cell line showed no effect when exposed to rhTRAIL.

### Expression of sfTRAIL

**Figure 1:**
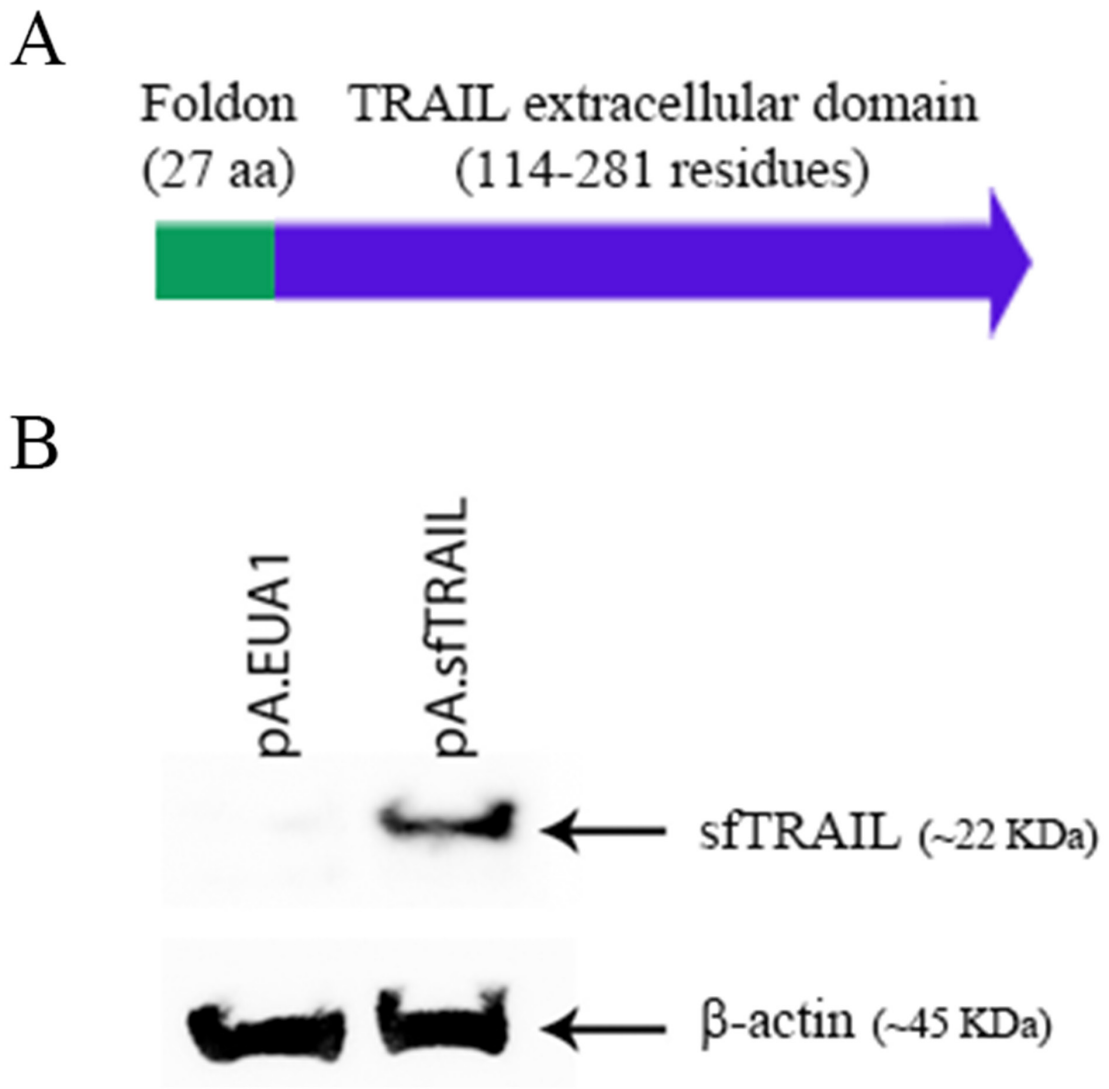
**(A)** Schematic representation of the sfTRAIL protein; **(B)** Western blot analysis of sfTRAIL protein expression. Vero cells were transduced with pA.EUA1 or pA.sfTRAIL amplicon vectors, at MOI1. Anti-TRAIL antibody and anti-β-actin were used.

*In silico* protein mass analysis (http://web.expasy.org/compute_pi/) indicates that the sfTRAIL protein constructed had a 22845.66 Da mass. To detect the sfTRAIL protein expression, we initially transduced Vero cells with pA.EUA1 or pA.sfTRAIL amplicon vectors. As expected, western blot results showed a band of ∼22 KDa, which represents the monomeric form of the sfTRAIL protein (Figure 1).

### Quantification of sfTRAIL in pA.sfTRAIL transduced Vero cells

Secretion of sfTRAIL protein was quantified by ELISA, in Vero cells transduced with pA.EUA1 or pA.sfTRAIL amplicon vector at MOI1. Subconfluent Vero cells were transduced in T75 cell culture flask with pA.EUA1 or pA.sfTRAIL amplicon vectors, at MOI1. Transduced Vero cell lines were able to produce filtered supernatants (SN) with a concentration of up to 1899.29 pg/mL (27.71 pM), which was the highest sfTRAIL production ever obtained by this method. Despite the concentration produced by this approach (about 2 ng/mL) not being enough for *in vivo* pre-clinical or clinical testing, it was sufficient for the cell culture assays performed, as shown below. Supernatants from pA.EUA1-transduced Vero cells were used as negative control, with undetectable production of TRAIL protein (Supplementary Figure 3).

### Bystander effect of sfTRAIL protein

**Figure 2:**
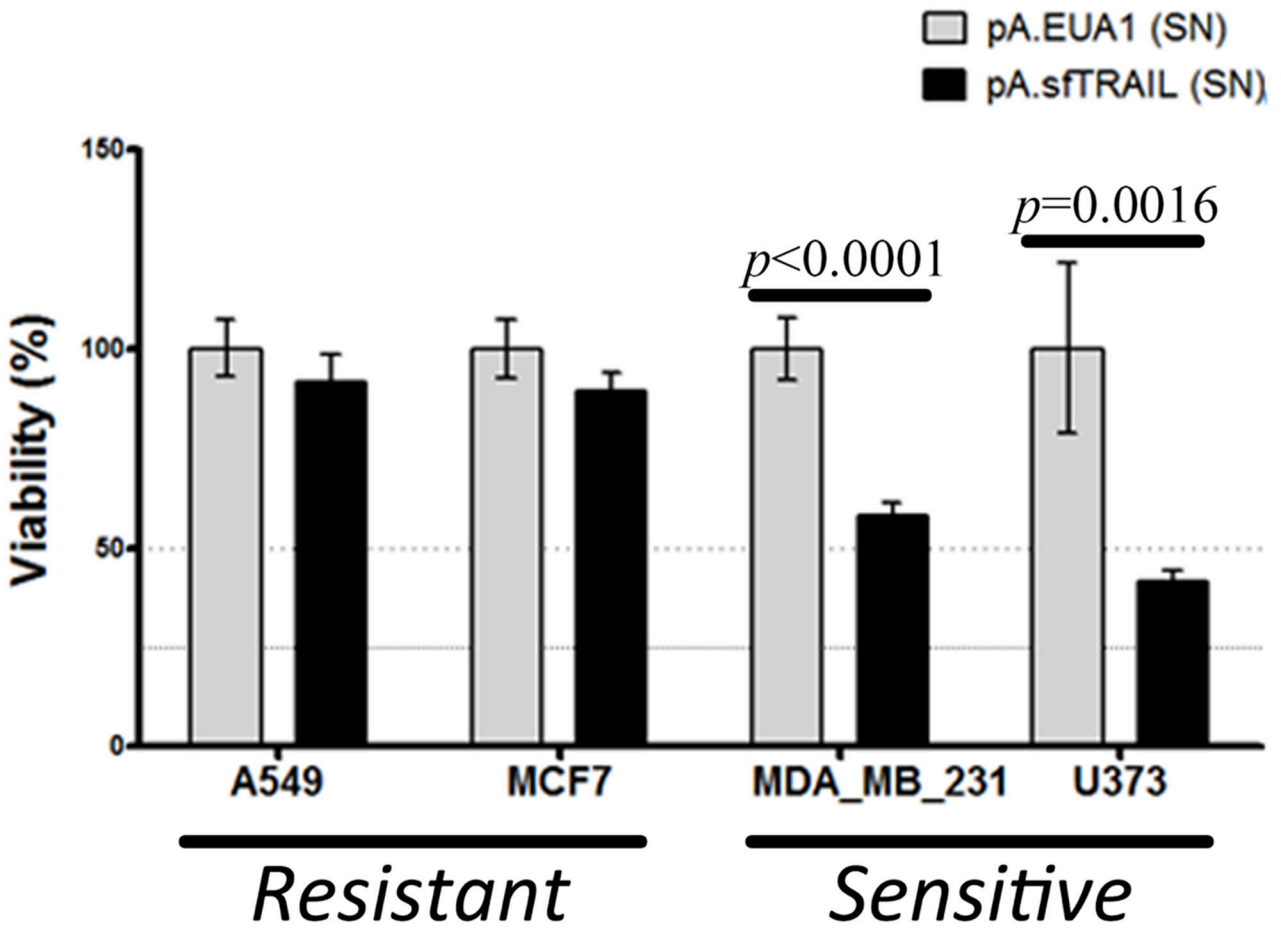
Cell viability (MTS) of cells incubated with sfTRAIL supernatants Values shown are the mean value (*±* SDs) of triplicates. (SN) means supernatants. sfTRAIL concentration in supernatants was 1899.29 pg/mL. Data represents three experiments performed in triplicate. p-value were assessed by Student’s *t*-test.

Next, we evaluated the capability of apoptosis induction of the sfTRAIL protein, using conditioned media from pA.sfTRAIL-transduced Vero cells described above, which sfTRAIL concentration was 1899.29 pg/mL (27.71 pM). Supernatants from pA.EUA1-transduced Vero cells were used as negative control. The MTS assay showed a statistically different viability reduction in both TRAIL-sensitive cell lines, U373 (p-value=0.0016) and MDA.MB.231 (p-value<0.0001), treated with sfTRAIL-containing supernatants (Figure 2). Statistical differences were not observed for TRAIL-resistant cell lines, A549 and MCF7.

### Apoptosis induction of sfTRAIL protein

**Figure 3:**
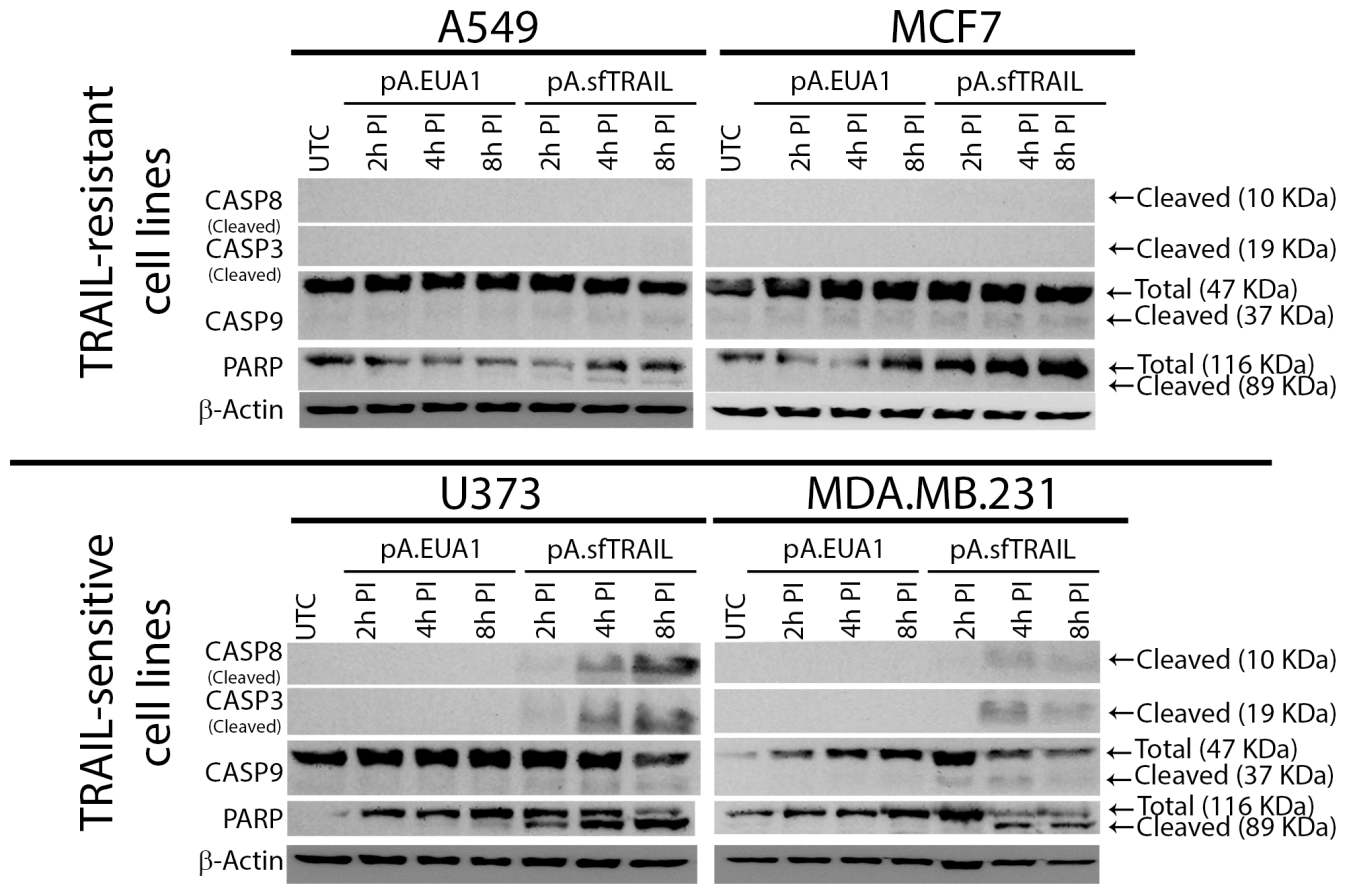
Western blot analysis of sfTRAIL-mediated apoptosis induction. sfTRAIL-mediated apoptosis induction of A549, MCF7, U373 and MDA-MB-231 cells, cultured in conditioned media, at 2h, 4h and 8h post-induction (PI) UTC, untreated control.

We further investigated the sfTRAIL protein pro-apoptotic properties. We also analyzed caspase-9 cleavage, which is one of the molecular components of the intrinsic mitochondrial apoptosis pathway, using filtered conditioned media from Vero transduced cells (Figure 3). Western blot analysis indicates cleavage of pro-caspase 8, pro-caspase 3 and PARP, upon induction with 0.22 μm-filtered conditioned media, produced in Vero cells transduced with pA.EUA1 or pA.sfTRAIL (1899.29 pg/mL; 27.71 pM). As expected, cleavage of pro-caspase 9 was not observed at these time points. In this way, sfTRAIL apoptosis induction rapidly activates the extrinsic apoptosis pathway, in TRAIL-sensitive cell lines, as early as 2 hours after sfTRAIL induction. We also observed a cleavage of caspase-9 in the MDA.MB.231 cell line treated with sfTRAIL-containing supernatants, which is consistent with its type II apoptotic behavior. We found that, sfTRAIL protein behaves as expected, promoting apoptosis at concentrations of no more than 2 ng/mL, in the sensitive cell lines used, but not in the resistant ones. At this point, it is worth to recall that we decided to include the analysis of caspase-3 cleavage in the MCF-7 cell line, despite the fact this cell line does not express this protein.

### Bystander effect of sfTRAIL protein at femtomolar concentrations

**Figure 4:**
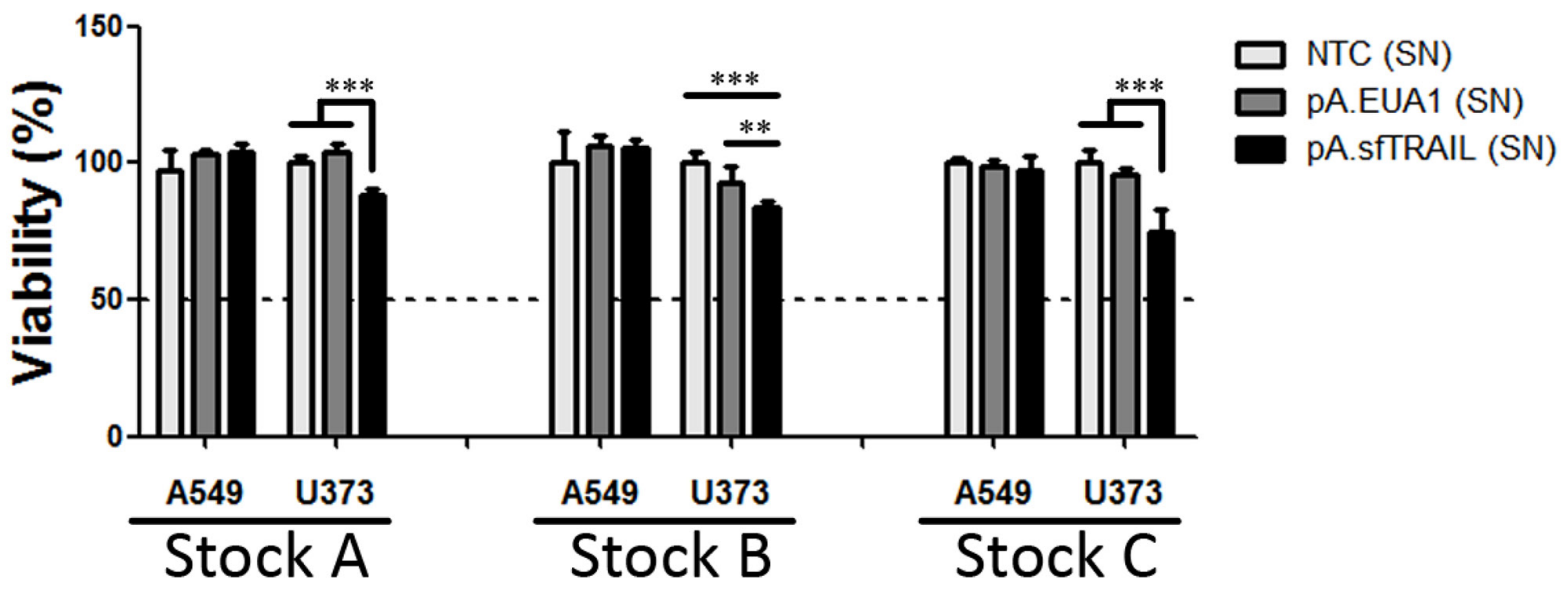
Cell viability (MTS) of cells incubated with sfTRAIL supernatants. Values shown are the mean value (± SDs) of three experiments performed in triplicate (Stock A, B and C) (SN) means supernatants; NTC (SN) represents supernatants of non-transduced cells; ^*^ indicates p-value (Student’s *t*-test), where ^**^ means p-value=0.0032 and ^***^ p-value<0.0001.

Next, we evaluated the ability to reduce cell viability of the sfTRAIL protein at femtomolar concentrations, using conditioned media from pA.sfTRAIL-transduced Vero cells described above, which sfTRAIL concentration was in mean 78.32 pg/mL. Supernatants from non-transduced cells (NTC) and pA.EUA1-transduced Vero cells were used as negative control. After 72 hours of incubation with conditioned supernatants, MTS assay showed a statistically different viability reduction (p-value=0.0032 or p-value<0.0001, depending on the supernatant stock used) in the U373 (TRAIL-sensitive) for all three protein productions, while the A549 (TRAIL-resistant) cell line did not respond to the sfTRAIL challenge (Figure 4). Thus, considering a trimeric sfTRAIL mass of 68.53698 KDa and a concentration of 68.45 pg/mL (sfTRAIL stock B), the sfTRAIL protein induced a decrease in cell viability at a concentration of 998.73 fM in U373 cells.

### Reproducibility of sfTRAIL protein production

In order to address the consistency between each sfTRAIL protein production, we transduced Vero cells with a EGFP-expressing amplicon control vector or the sfTRAIL-expressing amplicon vector in a biological triplicate. Transductions were done at MOI0.1. As a result, after the ELISA quantification we obtained sfTRAIL protein supernatants with a mean concentration of 78.32 pg/mL (Table 1 and Supplementary Figure 4). Variation between the three independent sfTRAIL protein productions was minimal, demonstrating a good reproducibility in the sfTRAIL production approach.

**Table 1:** ELISA quantification of sfTRAIL protein in non-transduced controls (NTC), pA.EUA1 or pA.sfTRAIL-transduced supernatants

Samples	Concentration (pg/mL)
**NTC_A**	-0.48
**NTC_B**	-0.68
**NTC_C**	-3.17
**pA.EUA1_A**	-2.34
**pA.EUA1_B**	-6.02
**pA.EUA1_C**	-6.97
**pA.TRAIL_A**	**84.80**
**pA.TRAIL_B**	**68.45**
**pA.TRAIL_C**	**81.71**

A, B and C identify the biological triplicate of the experiment

## DISCUSSION

The use of soluble TRAIL has been pronounced as an ideal therapeutic molecule exhibiting a selective and potent antitumor effect [8]. TRAIL-induced apoptosis leaves non-tumoral cells unharmed, underlying its potential as a therapeutic protein in oncology treatment. While this oncotargeted potential is poorly understood, it has encouraged the development of clinical trials of recombinant human TRAIL proteins. Among the tumor types already tested in clinical trials, Non-Small Cell Lung Cancer (Phase II), Colorectal Cancer (Phase I) and Non-Hodgkin’s Lymphomas (Phase I). However, the instability of TRAIL protein and its high cost constitute major drawbacks to its clinical use.

Soluble TRAIL (sTRAIL) stabilization as a trimer is essential for its binding to the effector TRAIL receptors, TRAIL-R1 and TRAIL-R2, even if expression of both TRAIL receptors were not described as predictors of TRAIL response [13, 14]. Several versions of recombinant soluble TRAIL with different N-terminal fusions domains have been reported [15–18]. Most TRAIL clinical trials use a non-tagged version, containing amino acids 114–281, stabilized by the addition of zinc and reducing agent to the cell-culture media and extraction buffers, and by formulation of the purified protein at neutral pH [19]. These further modifications increases the production steps and time, and may explain the high cost of the therapeutic protein. In addition, further modifications may affect the pro-apoptotic capabilities of the resulting trimer, requiring high drug concentrations.

To our knowledge, this is the first study describing the stabilization of TRAIL protein with a synthetic N-terminal fusion domain based on the C-terminal Foldon domain of the Fibritin T4 bacteriophage. This recombinant protein (sfTRAIL) was produced in cell culture supernatants of transfected/transduced Vero cells. Although Vero cells are not ideal for secreted recombinant protein production, this approach allowed a rapid proof-of-concept validation. Therefore, it is worth to note that sfTRAIL protein concentration may be further improved.

Regardless of the amount of protein produced, sfTRAIL protein was able to induce apoptosis at picomolar concentrations, and reduction in cell viability at femtomolar concentrations, without any special biochemical stabilization requirement. In this work, we produced recombinant TRAIL proteins simple by filtering cell culture supernatants. This may represent a major advantage over currently used recombinant TRAIL proteins available, even reducing its toxic side effects and improving patient outcome. Bioactive concentrations of current available soluble TRAIL variants are usually tested at concentrations of ng/mL or even μg/ml [10, 20–22].

The antitumor capabilities of the recombinant TRAIL variants here described were tested in four cancer cell lines with known TRAIL-sensitivity/resistance profiles, A549 (resistant), MCF7 (moderate-sensitive), U373 (sensitive) and MDA.MB.231. Although the MCF7 cell line was previously described as resistant, in our work it showed moderate sensitivity [10, 23]. So far, the bystander effect, transferred in the conditioned medium, was observed in the U373, MDA.MB.231 and MCF7 cell lines, not affecting the A549 cell line.

In conclusion, we showed that sfTRAIL protein exhibit a potent pro-apoptotic effect, triggering cell death at pico and femtomolar concentrations. In addition, as it is not necessary to further purify the supernatants, its low production requirement should reduce its final cost.

## MATERIALS AND METHODS

### Cell culture

Vero (African green monkey kidney), Vero-7b (Vero-derived cell line expressing ICP4/ICP27) [24], Gli36 (glioblastoma) were kindly provided by Dr. Alberto Epstein [25]. A549 (lung cancer) cell line was obtained from ECACC. Jurkat (leukemia), MCF7 (breast cancer), MDA.MB.231 (breast cancer) and HeLa (cervical cancer) cell lines were obtained from ATCC. U373 (glioma) cell line was kindly given by Dr. Joseph Costello. Cell lines were cultured in Dulbecco’s minimum essential medium (DMEM; Invitrogen, USA) supplemented with 10% fetal bovine serum (FBS; Invitrogen, USA), and 1% antibiotic-antimycotic solution (Invitrogen, USA). Vero-7b cells were selected with 1 mg/mL of G418 (Sigma-Aldrich, USA) at every three passages. All cell lines were maintained in a humidified 37°C atmosphere of 95% air and 5% CO_2_.

### Cell line authentication

Authentication of A549, MCF7, U373 and MDA.MB.231 cell lines was performed by short tandem repeat (STR) DNA typing according to the International Reference Standard for Authentication of Human Cell Lines using a panel of 8 (D5S818, D13S317, D7S820, D16S539, vWA, TH01, TPOX and CSF1P0) STR loci plus gender determination (AMEL), using the fluorescent labeling primers as reported by Dirks et al [26]. Briefly, 50ng of DNA was amplified in a multiplex PCR reaction carried out in a total volume of 10uL with the Qiagen multiplex kit (Qiagen) comprising 0.5 μM of all fluorescent primer pairs plus 1 μM of TH01 primer reinforcement. DNA amplification products were diluted 1:100 in ultrapure nuclease free water (USB, Cleveland, USA) and combined with 0.3 μL of the internal size standard 500ROX (Applied Biosystems, Foster City, CA, USA) in 8.7 μL of formamide and loaded automatically in a capillary electrophoresis system using the Genetic Analyzer ABI PRISM 3500 (Applied Biosystems). The analysis was performed in the GeneMapper software version 4.1 (Applied Biosystems). Genotyping confirmed the identity of all cell lines, with the exception of U373, which was shown to be a sub-clone of U251 cell line.

### Plasmid construction

cDNA from Jurkat cells was used to amplify the extracellular domain of the human TRAIL (aa 114-281). Total RNA was obtained from 1×10^6^ cells and converted in cDNA using RevertAid H Minus Reverse Transcriptase (Thermo Scientific) and oligo(dT)18 primer. The resulting cDNA was subsequently PCR amplified with the Phusion High-Fidelity DNA Polymerase (Thermo Scientific) using the primers sTRAIL_F and sTRAIL_R (Table 1) and the 550 bp PCR fragment was gel-purified, digested with EcoRI (Thermo Scientific) and ligated with T4 DNA ligase (Thermo Scientific) at the EcoRI restriction site of pCI.Neo plasmid (Promega), generating the pCI.Neo.sTRAIL plasmid.

The coding sequence of the Fibritin foldon domain, of the T4 Bacteriophage, was humanized using Sequence Manipulation Suite [27]. Both Foldon_F and Foldon_R ssDNA oligos were hybridized and ligated into the NheI site of pCI.Neo.sTRAIL, with T4 DNA ligase (Thermo Scientific). The resulting plasmid, named pCI.Neo.sfTRAIL, was sequenced to confirm the correct fusion gene structure. Escherichia coli DH5α cells (New England Biolabs, USA) were used for cloning experiments and plasmid propagation. Bacterial strains were routinely grown at 37°C in Luria-Broth (Sigma-Aldrich) or LB-agar (Sigma-Aldrich) containing medium and supplemented with 100 μg/mL ampicillin (Sigma).

### HSV-1-derived amplicon vector production

Amplicon plasmid pA.EUA1 [28] containing one HSV-1 replication origin and one HSV-1 package signal “a” was used to derive the pA.sfTRAIL amplicon plasmid. Firstly, pCI.Neo.sfTRAIL was digested with AseI and DraI (New England Biolabs), generating an 1804 bp fragment. After being blunt-ended with the Klenow enzyme (Thermo Scientific), this 1804 bp AseI/DraI fragment was subcloned into the SnaBI (New England Biolabs) site of the pA.EUA1 plasmid. The resulting plasmid, named pA.sfTRAIL, was used to produce amplicon vectors, as described below.

The defective cre-loxP-based helper virus, HSV-1 LaLΔJ [25] was used as helper to produce amplicon vectors that were propagated and titrated in Vero-7b cells. Amplicon vector stocks were produced in T25 flasks containing 3×10^6^ Vero-7b cells, previously transfected with pA.EUA1 or pA.sfTRAIL plasmids, infected at multiplicity of infection (MOI) of 0.1 plaque forming unit (PFU)/cell in Medium 199 (Invitrogen, USA) supplemented with 1% FBS (M199 1% FBS). When complete cytophatic effect (CPE) was observed (48∼72 h post-infection), viruses were harvested. Thus, infected cells were centrifuged at 1,000 × g for 10 min at 4°C. The formed pellet was diluted in 400 μL of M199 1% FBS and frozen/thawed three times to break down the infected cells and facilitate the viral particles release. The pellet solution was clarified at 1,000 × g for 10 min at 4°C, aliquoted and stored at -80°C until use. Amplicon/helper particles were tittered by plaque assay [25] in Gli36 and Vero-7b cells respectively.

### Western blot analysis

Vero cells were seeded in 6 well plate at a density of 5×10^5^ cells per well. Twenty-four hours later, cells were transduced with pA.EUA1 or pA.sfTRAIL amplicon particles at MOI1. After 48 h, supernatants and cells were collected separately. Supernatants were filtered in 0.22 μm filters. Cells were rinsed in ice-cold PBS then scraped and lysed in lysis buffer (50mM Tris pH7.6–8, 150mM NaCl, 5mM EDTA, 1mM Na3VO4, 10mM NaF, 10mM sodium pyrophosphate, 1% NP-40, and protease cocktail inhibitors). Western blotting was performed using standard SDS-PAGE gel, loading 20 μg of protein per lane. The antibodies used were: rabbit polyclonal anti-TRAIL (abcam, #ab2056), rabbit monoclonal anti-caspase 8 (Cell Signaling, #4790), rabbit polyclonal anti-caspase 3 (Cell Signaling, #9662), mouse monoclonal anti-caspase 9 (Cell Signaling, #9508), rabbit polyclonal anti-PARP (Cell Signaling, #9542) and β-actin (Cell Signaling, #3700). After incubation with the appropriate horseradish peroxidase (HRPO)-conjugated secondary antibody (Cell Signaling, dilution 1:5000), the immunoreactivity was visualized by chemiluminescence (Amersham ECL Western Blotting Detection Reagent, GE Healthcare).

### Electroporation

Electroporations of 8 × 10^6^ HeLa cells were carried out with two pulses of 200 V and 850 μF, in 2 mm cuvettes, using Gene Pulser II (Bio-rad). Electroporated cells were seeded in T75 cell culture flasks and further maintained in a humidified 37°C atmosphere with 5% CO_2_. After 48 hours of incubation, supernatants were cleared by centrifugation followed by filtration with 0.22 μm filters and stored at 4°C.

### ELISA protein quantification

sfTRAIL protein contained in filtered supernatants were quantified by ELISA, using Human TRAIL/TNFSF10 Quantikine ELISA kit (R&D Systems, Minneapolis, MN, USA), following the manufacturer’s instructions. Serially diluted rhTRAIL (R&D Systems, Minneapolis, MN, USA) was used for indirect quantification. It is worth to note that along the experiments, sfTRAIL protein was produced in two opportunities. Protein quantification was performed by ELISA at each sfTRAIL production.

### Cell viability assay

To determine the effect of sfTRAIL protein in cellular viability over time, A549, MCF7, MDA.MB.231 and U373 cells were seeded into 96-well plates, at a concentration of 5 × 10^3^ cells per well, as previously described [29]. After 24 hours, cells were cultured in 0.22 μm-filtered conditioned media. After 48 hours of incubation, viable cells were quantified using MTS assay (CellTiter 96® AQueous One Solution Cell Proliferation Assay, Promega). Assays were performed in triplicate and repeated three times. In order to analyze the stability of the sfTRAIL protein, filtered supernatants were maintained at 4°C during 60 days, without any conservator. After this period, all four cell lines were rechallenged to TRAIL induced apoptosis and assayed by MTS analysis.

### Apoptosis and TRAIL receptors detection by flow cytometry

For apoptosis analysis, cells were incubated with rhTRAIL (R&D Systems, Minneapolis, MN, USA) at 100 ng/mL for 4 hours. After incubation, cells were washed in cold PBS and apoptosis was assessed by flow cytometry using the Annexin V-PE/7-AAD apoptosis detection kit (BD Biosciences), according to the manufacturer’s protocol. Briefly, 10^5^ cells in 100 μL of 1x binding buffer were incubated with 5 μL Annexin V-PE and 5 μL 7-AAD reagent for 15 minutes, at room temperature in the dark. After incubation 400 μL of 1x binding buffer were added and tubes were directly analyzed in a FACS Aria II (BD Bioscience).

Expression of TRAIL receptors was also evaluated by flow cytometry. Thus, incubation with antibodies was performed as described above, using human anti-DR4-PE (R&D Systems - Clone 69036) and anti-DR5-PE (R&D Systems - Clone 71908) antibodies. Non-stained cells were used as controls, setting gates at 1% positivity for comparisons.

### Statistical analysis

The differences between groups were evaluated by calculating Student’s *t*-test, one-way ANOVA with post hoc comparison Tukey’s test. For all tests, p-value < 0.05 were considered statistically significant.

## SUPPLEMENTARY MATERIALS FIGURES



## References

[1] Savill J, Fadok V (2000). Corpse clearance defines the meaning of cell death. Nature.

[2] Ashkenazi A, Holland P, Eckhardt SG (2008). Ligand-based targeting of apoptosis in cancer: the potential of recombinant human apoptosis ligand 2/Tumor necrosis factor-related apoptosis-inducing ligand (rhApo2L/TRAIL). J Clin Oncol.

[3] Wiezorek J, Holland P, Graves J (2010). Death receptor agonists as a targeted therapy for cancer. Clin Cancer Res.

[4] Holland PM (2014). Death receptor agonist therapies for cancer, which is the right TRAIL?. Cytokine Growth Factor Rev.

[5] Hymowitz SG, O’Connell MP, Ultsch MH, Hurst A, Totpal K, Ashkenazi A, de Vos AM, Kelley RF (2000). A unique zinc-binding site revealed by a high-resolution X-ray structure of homotrimeric Apo2L/TRAIL. Biochemistry.

[6] Verbrugge I, Johnstone RW, Smyth MJ (2010). SnapShot: Extrinsic apoptosis pathways. Cell.

[7] LeBlanc HN, Ashkenazi A (2003). Apo2L/TRAIL and its death and decoy receptors. Cell Death Differ.

[8] Ashkenazi A, Pai RC, Fong S, Leung S, Lawrence DA, Marsters SA, Blackie C, Chang L, McMurtrey AE, Hebert A, DeForge L, Koumenis IL, Lewis D (1999). Safety and antitumor activity of recombinant soluble Apo2 ligand. J Clin Invest.

[9] Jin CY, Moon DO, Lee JD, Heo MS, Choi YH, Lee CM, Park YM, Kim GY (2007). Sulforaphane sensitizes tumor necrosis factor-related apoptosis-inducing ligand-mediated apoptosis through downregulation of ERK and Akt in lung adenocarcinoma A549 cells. Carcinogenesis.

[10] Zhang Y, Zhang B (2008). TRAIL resistance of breast cancer cells is associated with constitutive endocytosis of death receptors 4 and 5. Mol Cancer Res.

[11] Yoshida T, Zhang Y, Rivera Rosado LA, Zhang B (2009). Repeated treatment with subtoxic doses of TRAIL induces resistance to apoptosis through its death receptors in MDA-MB-231 breast cancer cells. Mol Cancer Res.

[12] Chen JJ, Knudsen S, Mazin W, Dahlgaard J, Zhang B (2012). A 71-gene signature of TRAIL sensitivity in cancer cells. Mol Cancer Ther.

[13] Plummer R, Attard G, Pacey S, Li L, Razak A, Perrett R, Barrett M, Judson I, Kaye S, Fox NL, Halpern W, Corey A, Calvert H (2007). Phase 1 and pharmacokinetic study of lexatumumab in patients with advanced cancers. Clin Cancer Res.

[14] Wakelee HA, Patnaik A, Sikic BI, Mita M, Fox NL, Miceli R, Ullrich SJ, Fisher GA, Tolcher AW (2010). Phase I and pharmacokinetic study of lexatumumab (HGS-ETR2) given every 2 weeks in patients with advanced solid tumors. Ann Oncol.

[15] Pitti RM, Marsters SA, Ruppert S, Donahue CJ, Moore A, Ashkenazi A (1996). Induction of apoptosis by Apo-2 ligand, a new member of the tumor necrosis factor cytokine family. J Biol Chem.

[16] Walczak H, Miller RE, Ariail K, Gliniak B, Griffith TS, Kubin M, Chin W, Jones J, Woodward A, Le T, Smith C, Smolak P, Goodwin RG (1999). Tumoricidal activity of tumor necrosis factor-related apoptosis-inducing ligand *in vivo*. Nat Med.

[17] Bodmer JL, Meier P, Tschopp J, Schneider P (2000). Cysteine 230 is essential for the structure and activity of the cytotoxic ligand TRAIL. J Biol Chem.

[18] Mohr A, Lyons M, Deedigan L, Harte T, Shaw G, Howard L, Barry F, O’Brien T, Zwacka R (2008). Mesenchymal stem cells expressing TRAIL lead to tumour growth inhibition in an experimental lung cancer model. J Cell Mol Med.

[19] Ashkenazi A, Dixit VM (1999). Apoptosis control by death and decoy receptors. Curr Opin Cell Biol.

[20] Hougardy BM, Reesink-Peters N, van den Heuvel FA, ten Hoor KA, Hollema H, de Vries EG, de Jong S, van der Zee AG (2008). A robust ex vivo model for evaluation of induction of apoptosis by rhTRAIL in combination with proteasome inhibitor MG132 in human premalignant cervical explants. Int J Cancer.

[21] Meijer A, Kruyt FA, van der Zee AG, Hollema H, Le P, ten Hoor KA, Groothuis GM, Quax WJ, de Vries EG, de Jong S (2013). Nutlin-3 preferentially sensitises wild-type p53-expressing cancer cells to DR5-selective TRAIL over rhTRAIL. Br J Cancer.

[22] Jalving M, de Jong S, Koornstra JJ, Boersma-van Ek W, Zwart N, Wesseling J, de Vries EG, Kleibeuker JH (2006). TRAIL induces apoptosis in human colorectal adenoma cell lines and human colorectal adenomas. Clin Cancer Res.

[23] Bozza WP, Tolleson WH, Rosado LA, Zhang B (2015). Ricin detection: tracking active toxin. Biotechnol Adv.

[24] Krisky DM, Marconi PC, Oligino T, Rouse RJ, Fink DJ, Glorioso JC (1997). Rapid method for construction of recombinant HSV gene transfer vectors. Gene Ther.

[25] Zaupa C, Revol-Guyot V, Epstein AL (2003). Improved packaging system for generation of high-level noncytotoxic HSV-1 amplicon vectors using Cre-loxP site-specific recombination to delete the packaging signals of defective helper genomes. Hum Gene Ther.

[26] Dirks WG, Faehnrich S, Estella IA, Drexler HG (2005). Short tandem repeat DNA typing provides an international reference standard for authentication of human cell lines. ALTEX.

[27] Stothard P (2000). The sequence manipulation suite: JavaScript programs for analyzing and formatting protein and DNA sequences. Biotechniques.

[28] Adrover MF, Guyot-Revol V, Cheli VT, Blanco C, Vidal R, Alche L, Kornisiuk E, Epstein AL, Jerusalinsky D (2003). Hippocampal infection with HSV-1-derived vectors expressing an NMDAR1 antisense modifies behavior. Genes Brain Behav.

[29] Silva-Oliveira RJ, Silva VA, Martinho O, Cruvinel-Carloni A, Melendez ME, Rosa MN, de Paula FE, de Souza Viana L, Carvalho AL, Reis RM (2016). Cytotoxicity of allitinib, an irreversible anti-EGFR agent, in a large panel of human cancer-derived cell lines: KRAS mutation status as a predictive biomarker. Cell Oncol (Dordr).

