# Supplementary material for: Construction and characterization of a new TRAIL soluble form, active at picomolar concentrations
Source: Oncotarget. 2018 Jun 5;9(43):27233–41. doi: 10.18632/oncotarget.25519 (PMC6007462; doi:10.18632/oncotarget.25519)
Supplement: Supplementary file 1 [file oncotarget-09-27233-s001.pdf]

Construction and characterization of a new TRAIL soluble form, active at picomolar concentrations

SUPPLEMENTARY MATERIALS

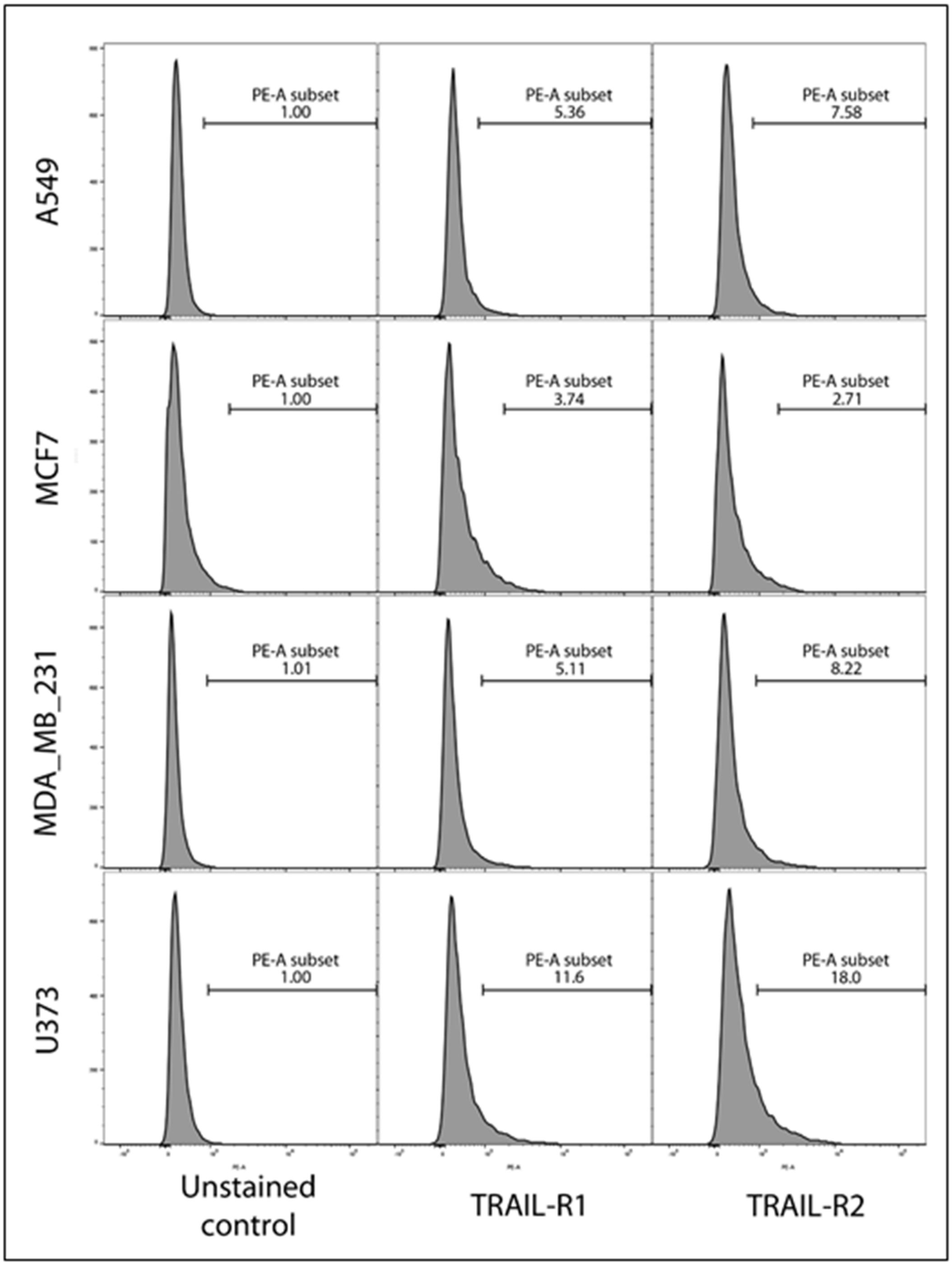

Supplementary Figure 1: Flow cytometry analysis of TRAIL receptors. Gates were fixed using unstained controls at 1% of positivity.

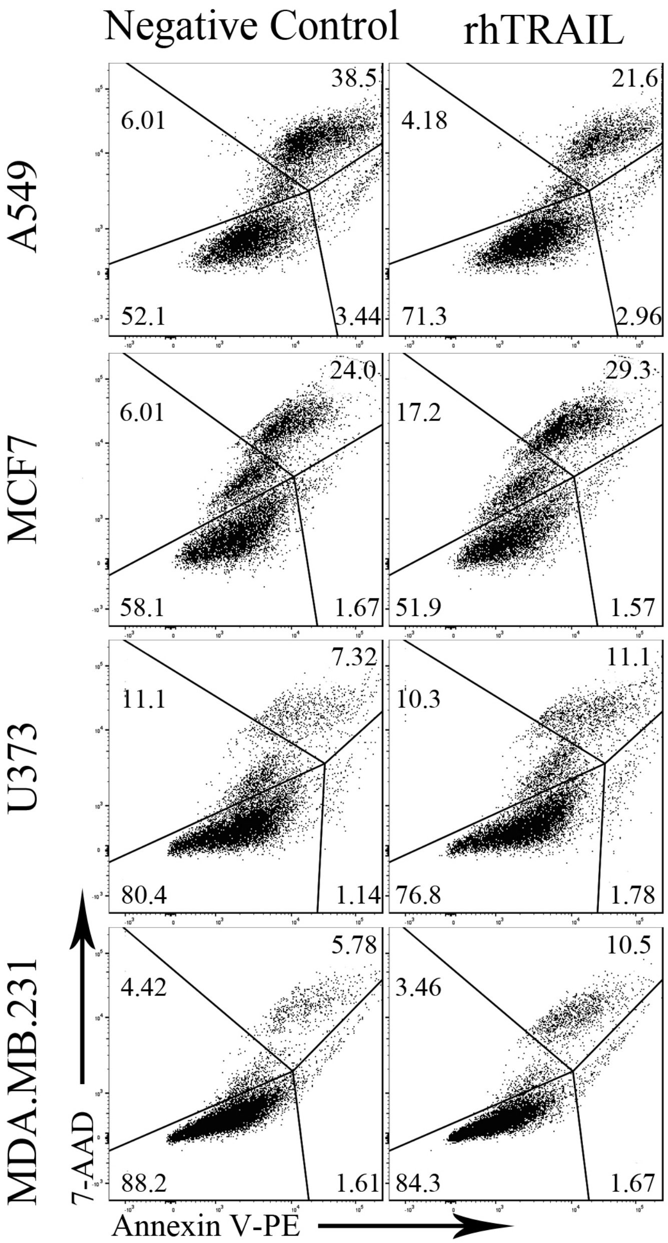

Supplementary Figure 2: Flow cytometry analysis of rhTRAIL-induced apoptosis. Numbers indicated in quadrants represent percentages.

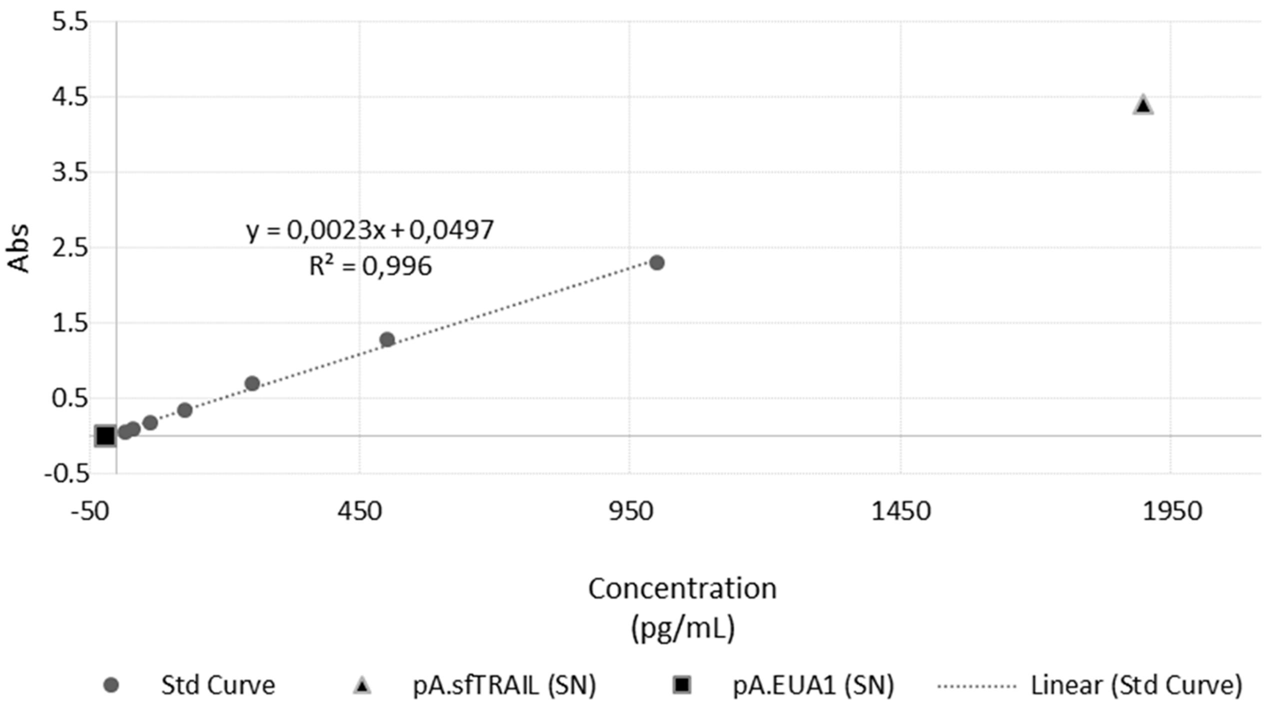

Supplementary Figure 3: Elisa quantification of sfTRAIL protein from transduced-cells supernatants. Vero cells were transduced with pA.EUA1 or pA.sfTRAIL amplicon vectors, at MOI1.

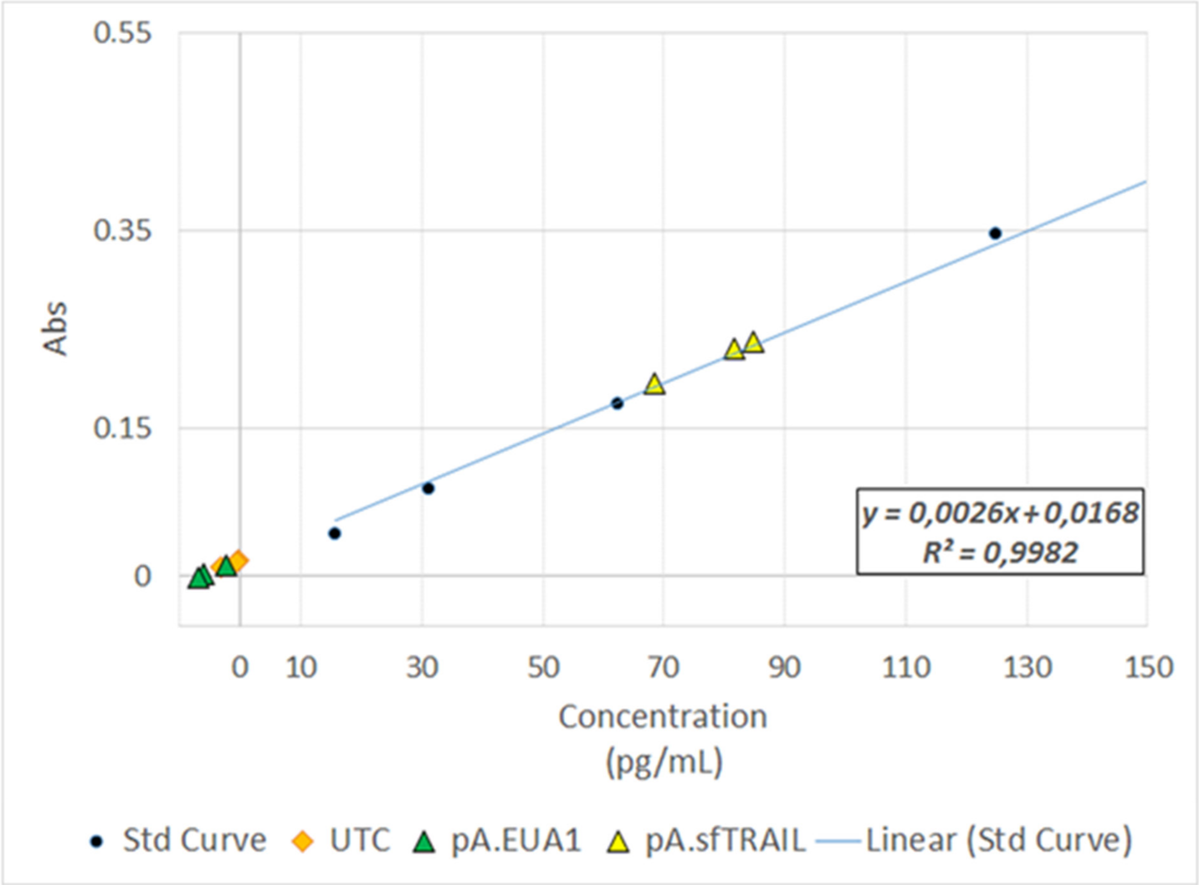

Supplementary Figure 4: Elisa quantification of sfTRAIL protein from transduced-cells supernatants. Vero cells were transduced with pA.EUA1 or pA.sfTRAIL amplicon vectors, at MOI0.1. Non-transduced cells were used as negative controls (UTC).
